# Transcriptomic Analysis Reveals AKT1 Upregulation in Inner Mongolian Cashmere Goats at 12 and 15 Months of Age

**DOI:** 10.3390/vetsci13070671

**Published:** 2026-07-10

**Authors:** Muhammad Zain Ghauri, Ayesha Zafar, M Khuzema Niaz, Usman Nazir, Asim Munir, Muhammad Hamza, Kiran Zahra, Dejun Ji

**Affiliations:** 1 College of Animal Science and Technology, Yangzhou University, Yangzhou 225009, China; zainghauri123@gmail.com (M.Z.G.); ayeshazafar4979@gmail.com (A.Z.); khuzemaniazmughal@gmail.com (M.K.N.); usmann539@gmail.com (U.N.); muhhamza357@hotmail.com (M.H.); kiranzahra1205@gmail.com (K.Z.); 2 College of Bioscience and Biotechnology, Yangzhou University, Yangzhou 225009, China; 3 Jiangsu Co-Innovation Center for Prevention and Control of Important Animal Infectious Diseases and Zoonoses, College of Veterinary Medicine, Yangzhou University, Yangzhou 225009, China; asimmuniruaf49@gmail.com

**Keywords:** cashmere goats, transcriptomic analysis, *AKT1* signaling pathway, hair follicle development, age-dependent gene expression

## Abstract

Cashmere production declines with age in *Inner Mongolian* cashmere goats, but the molecular mechanisms are unknown. This study employed a transcriptome-wide RNA-seq analysis to compare age-associated and breed-related gene expression profiles in goat skin, with particular emphasis on age-dependent *AKT1* expression and its associated signaling pathways. Skin tissues from cashmere goats at 12 and 15 months and age-matched non-cashmere controls were analyzed by RNA-seq (BGIDNBSEQ platform). Differential expression and functional enrichment analyses were performed on skin transcriptomes of cashmere and control goats at 12 and 15 months. Age-specific functional profiles emerged: 12-month cashmere goats showed enrichment in GTPase activity, glucose transport, and ribosomal assembly, whereas 15-month goats were enriched for autophagosome assembly and apoptosis. FoxO signaling was commonly enriched across both ages, while the Hepatitis B pathway was unique to 12 months. *AKT1* was significantly upregulated at 12 months coinciding with peak cashmere production with reduced expression at 15 months. This age-dependent pattern was supported by coordinated regulation of downstream effectors, including *TSC2*, *PIK3R2*, *MAPK9*, and *FOXO3*. Collectively, these transcriptomic data provide a mechanistic framework linking age-dependent *AKT1* regulation to the decline in cashmere fiber production.

## Simple Summary

This study investigates the effect of age on cashmere fiber production in Inner Mongolian cashmere goats by analyzing skin transcriptomic profiles of 12- and 15-month-old cashmere goats, with non-cashmere goats included as a reference group. The results indicate that both age and breed contribute substantially to variation in gene expression patterns related to hair follicle development and cashmere fiber production. One of the key findings was the higher activity of the *AKT1* gene in younger goats, which plays an important role in controlling cell growth, survival, and metabolism, all of which are essential for healthy hair follicle function and high-quality fiber production. In younger goats, genes linked to growth, protein production, and energy metabolism were more active, which helps to explain their better cashmere yield and quality. In contrast, older goats showed increased activity in genes associated with stress, cell breakdown, and aging processes such as autophagy and apoptosis. These changes may contribute to the decline in fiber production as goats age. Overall, this study provides useful insight into the biological mechanisms behind cashmere production and highlights important genes and pathways that could be targeted in breeding programs to improve fiber quality and yield.

## 1. Introduction

The natural fiber known as cashmere is produced by cashmere goats (*Capra hircus*). Because of its exceptional softness, beautiful texture, and superior insulating properties, it is highly valued [1]. The production of this fiber greatly contributes to the livelihoods of pastoral people. The recent concentration of the global cashmere industry can be attributed to China’s rise to prominence in both raw fiber production and processing. Cashmere, a natural fiber produced by cashmere goats (*Capra hircus*), is highly valued for its softness, fine texture, and excellent insulating properties [1]. It is economically important for pastoral communities, with China playing a leading role in global production and processing. Among Chinese breeds, the Inner Mongolian cashmere goat is an important genetic resource due to its high fiber yield and quality. Therefore, sustainable breeding should aim to improve fiber output and quality while maintaining adaptation to harsh environmental conditions [2].

From a biological perspective, the “double-coat” system that creates cashmere fibers is composed of two distinct types of follicles. Primary follicles produce the coarse outer hairs that provide protection, while secondary follicles produce the delicate undercoat fibers that comprise cashmere. Cashmere production in goats is primarily determined by secondary hair follicle density, the ratio of secondary to primary follicles, and the activity of follicle cycles (anagen, catagen, and telogen) [3]. These dynamic cycles are orchestrated by intricate interactions between progenitor cells, epidermal stem cells, and the dermal papilla key regulatory hub for follicle formation and regeneration. The highly synchronized and seasonal follicle cycling across the body makes cashmere goats an exceptional model for investigating the molecular drivers of fiber production [4]. At the molecular level, hair follicle growth and cycling are governed by conserved signaling networks, including Wnt/β-catenin, BMP/TGF-β, Hedgehog, and Notch, alongside metabolic regulators such as MAPK and PI3K–AKT [5]. Within this network, AKT1 (Protein Kinase B) acts as a central signaling hub, phosphorylating downstream effectors including FOXO, TSC2, and GSK3β to coordinate cellular proliferation, survival, and nutrient responses [6].

*AKT1* is particularly critical for keratinocyte proliferation and differentiation, with genetic perturbations in this axis consistently associated with altered hair length phenotypes across mammalian species [7]. Recent transcriptomic efforts in cashmere goats have largely concentrated on seasonal photoperiod responses, embryonic follicle development, and single-cell heterogeneity of dermal papilla cells, uncovering breed-specific expression patterns and dynamic regulatory networks across developmental stages [8,9].

However, despite the well-established decline in cashmere quality and yield with advancing age, age-dependent transcriptional changes in goat skin remain conspicuously understudied [10,11]. Specifically, the transition from 12 to 15 months of age in Inner Mongolian cashmere goats represents a critical window during which fiber production noticeably diminishes, yet the underlying molecular mechanisms are largely unknown [12,13,14]. Goats’ age has a crucial role in the production of fiber; younger goats usually yield finer and better-quality cashmere. An attractive target for examining age-related alterations in hair follicle biology is the AKT1–FOXO3 signaling axis.

To address this gap, we performed comparative transcriptome sequencing of skin tissues from cashmere and non-cashmere (control) goats at 12 and 15 months of age. Our objective was to identify age-associated transcriptional signatures, with a focused interpretation of the *AKT1* signaling axis and its downstream regulators, thereby providing mechanistic insights into the age-related deterioration of cashmere fiber production [15,16,17,18].

## 2. Materials and Methods

### 2.1. Animal Ethics Statement

All animal procedures were approved by the IACUC of Yangzhou University (No. 202503133), following ARRIVE guidelines. All procedures were performed in accordance with institutional animal welfare laws to minimize suffering.

### 2.2. Experimental Animals and Skin Sample Collection

The study included two goat populations: Chinese Yangtze River Delta White goats (P) and Inner Mongolian cashmere goats (K). Four cashmere goats were selected, including R3 and R4 at 12 months of age and C1 and C2 at 15 months of age. In addition, four Chinese Yangtze River Delta White goats (P1–P4), aged 10–12 months, were included in the experiment. All animals were maintained under conventional farm-management conditions by a local farmer at a commercial goat breeding farm in Gaoyou, Yangzhou City, Jiangsu Province, China. Sample collection was conducted with the farm owner’s consent and in compliance with institutional ethical guidelines.

Skin biopsy specimens were acquired utilizing normal veterinary dermatological techniques for transcriptome analyses of animal integument. A 5 cm × 5 cm hair patch was excised from the caudal aspect of the left scapula, specifically at the level of the scapular spine and approximately 2 cm lateral to the dorsal midline. Residual hair was removed with a razor, and the exposed skin was sterilized with alcohol and iodine. A sterile 1 cm circular biopsy punch was then employed to obtain full-thickness skin tissue.

To control hemorrhage, a sterile topical hemostatic powder was applied to the biopsy site. The harvested skin samples (1 cm^2^ each) were immediately snap-frozen in liquid nitrogen and preserved at −80 °C until RNA extraction for transcriptome sequencing. In total, four skin samples were collected from Chinese Yangtze river delta white goats and four from cashmere goats.

### 2.3. RNA Extraction and Quality Assessment

Total RNA was extracted from approximately 30 mg of skin tissue using TRIzol™ reagent (Invitrogen RC112; Vazyme Biotech Co., Ltd., Nanjing, China) following the manufacturer’s instructions. RNA concentration and purity were evaluated with a NanoDrop^TM^ 2000 spectrophotometer (Thermo Fisher Scientific, Waltham, MA, USA). The absorbance ratios at 260/280 nm and 260/230 nm were used to assess RNA purity and potential contamination.

### 2.4. Library Preparation, Transcriptome Sequencing, and Bioinformatic Analysis

RNA samples were used to construct poly(A)-enriched, strand-specific transcriptome libraries. Messenger RNA was first captured with oligo(dT) magnetic beads and then fragmented into short RNA segments. First-strand complementary DNA (cDNA) was synthesized using random primers, followed by second-strand synthesis with dUTP incorporation to preserve transcript strand information. The resulting cDNA fragments were subjected to end repair, adenylation at the 3′ ends, adapter ligation, uracil DNA glycosylase treatment, and PCR enrichment. Qualified libraries were circularized to generate single-stranded circular DNA molecules and subsequently amplified into DNA nanoballs. Sequencing was carried out on the BGI-DNBSEQ platform using paired-end 150 bp reads. After sequencing, raw reads were filtered to remove low-quality sequences and then mapped to the goat reference genome. Gene expression was quantified as raw read counts. Principal component analysis (PCA) was performed using variance-stabilized normalized count data to evaluate sample distribution and clustering patterns. Differentially expressed genes were identified with the DESeq2 package. Genes meeting the defined absolute log_2_ fold-change threshold and a Benjamini–Hochberg adjusted *p*-value of less than 0.05 were considered significantly differentially expressed. Functional interpretation of these genes was performed through Gene Ontology (GO) and Kyoto Encyclopedia of Genes and Genomes (KEGG) enrichment analyses. Multiple-testing correction was applied using the Benjamini–Hochberg method, and enriched terms with a false discovery rate (FDR) q-value below 0.05 were regarded as statistically significant.

### 2.5. Gene Expression Analysis by qRT-PCR

To verify the reliability of the RNA-seq results, selected differentially expressed genes were validated by quantitative real-time PCR (qRT-PCR) using RNA isolated from the corresponding biological tissues. RNA concentration and purity were assessed with a NanoDrop 2000 spectrophotometer (Thermo Fisher Scientific, Waltham, MA, USA). For cDNA synthesis, 1 μg of total RNA was reverse transcribed using the PrimeScript RT Reagent Kit with gDNA Eraser (Takara Bio, Los Angeles, CA, USA).

qRT-PCR was performed with TB Green Premix Ex Taq II (Tli RNase H Plus; Takara Bio, Los Angeles, CA, USA) on a Bio-Rad CFX384 real-time PCR system, according to the manufacturer’s instructions and the previously described protocol [19]. Relative gene-expression levels were calculated using the 2^^(−ΔΔCt)^ method, with β-actin used as the internal reference gene [1]. The primer sequences used for qRT-PCR analysis are presented in Table 1. All primers were designed and synthesized by Azenta Life Sciences Co., Ltd. (Burlington, MA, USA).

### 2.6. Statistical Analysis

RNA-seq differential expression was analyzed using DESeq2 with significance thresholds of |log_2_FC| > 1 and Benjamini–Hochberg adjusted q < 0.05. Principal component analysis and hierarchical clustering were performed in R to assess sample relationships. Gene Ontology and KEGG pathway enrichment were evaluated using clusterProfiler with q < 0.05. qRT-PCR data were analyzed by the 2^^(−ΔΔCt)^ method and two-tailed Student’s *t*-test (mean ± SEM, *p* ≤ 0.05).

## 3. Results

### 3.1. Identification of DEGs

**Figure 1:**
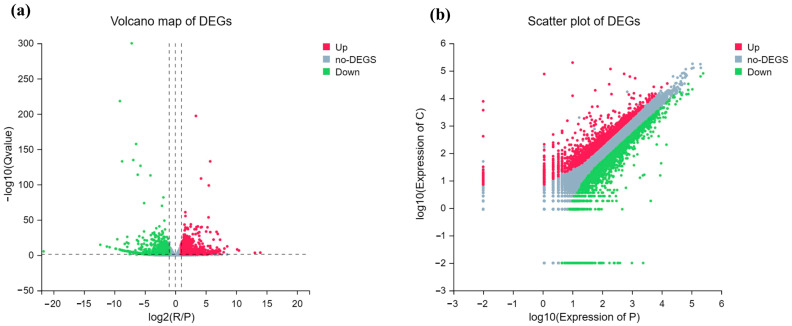
Differential expression analysis between R and P conditions. (**a**) Volcano plot showing log_2_ fold change vs. −log_10_(Q-value). (**b**) Scatter plot comparing expression levels in P and C. Red, gray, and green dots represent upregulated, non-differentially expressed, and downregulated genes, respectively.

To find DEGs between cashmere goats and Chinese Yangtze river delta white goats, stratified by age (R/P and C/P comparisons), a thorough transcriptome analysis was carried out. Different expression profiles were shown by the scatter plot (Figure 1b) and volcano map (Figure 1a), with several genes exhibiting significant upregulation and downregulation according to the imposed criteria (|log_2_(Fold Change)| > 1 and Benjamini–Hochberg adjusted q < 0.05; note that in the volcano plot, this adjusted threshold corresponds to −log_10_(q) > 1.3). The scatter plot highlighted the dispersion of highly variable genes and provided further insight into the overall expression correlation between samples. The number of DEGs varied significantly between the younger (R/P) and older (C/P) age groups, indicating an age-dependent component to the differential expression. Raw data of transcriptomic analysis is given in Supplementary Material (Tables S1–S4). These results show a substantial transcriptional response linked to the cashmere phenotype (Figure 1).

### 3.2. Clustering and Principal Component Analysis of DEGs

**Figure 2:**
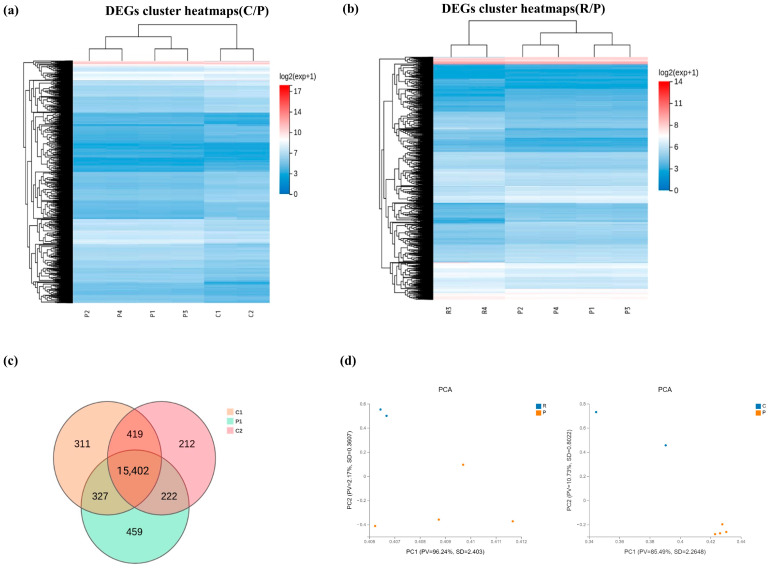
Validates experimental design through clustering and PCA. Panel (**a**) shows 15-month cashmere goats (C1–C2) clustering separately from controls (P1–P4), confirming distinct transcriptional profiles. Panel (**b**) shows 12-month cashmere goats (R3–R4) also clustering separately from controls, with a wider dynamic range indicating more pronounced changes in younger goats. Panel (**c**) presents a Venn diagram showing 247 unique DEGs in C vs. P, 315 unique in R vs. P, and 128 common to both. Panel (**d**) shows PCA with clear separation of all three groups along PC1 (42%) and PC2 (23%), confirming distinct transcriptional states.

The cashmere goat groups and the Chinese Yangtze river delta white group (P1–P4) were clearly separated by hierarchical clustering heatmaps of DEGs for both the C/P and R/P comparisons. In contrast to the Chinese Yangtze river delta white goat samples, the cashmere goat samples (C1, C2, and R3, R4) clustered together, indicating reliable and consistent expression patterns within each phenotypic. These classifications were further confirmed using principal component analysis (PCA); the first two principal components accounted for a significant amount of the variance (e.g., PC1 = 96.24% for C/P comparison). The cashmere samples formed a compact cluster away from the controls, and the PCA plots demonstrated a clear difference between the R and P groups as well as between the C and P groups (Figure 2).

### 3.3. Gene Ontology Enrichment Analysis

**Figure 3:**
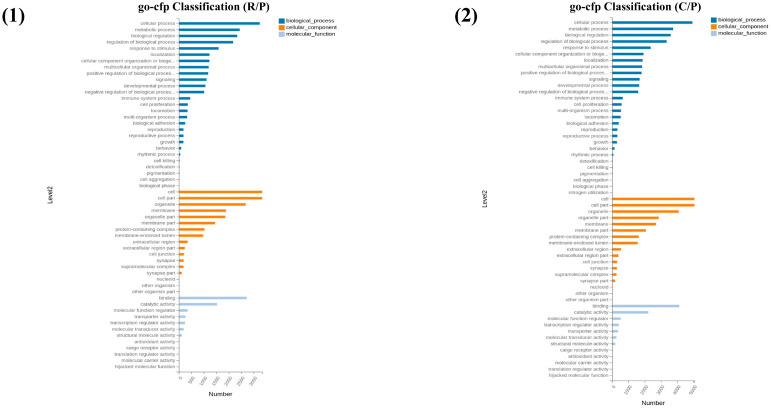
(**1**) Level 2 classification for R vs. P, with top terms including cellular process, metabolic process, and biological regulation for Biological Process; cell, cell part, and organelle for Cellular Component; and binding and catalytic activity for Molecular Function. (**2**) Level 2 classification for C vs. P with remarkably consistent distribution, suggesting fundamental biological processes are similarly affected across age groups.

As shown in Figure 3, Different functional themes between the two age comparisons were identified by GO biological process enrichment analysis. Translation, apoptotic process, negative regulation of fibroblast proliferation, and sphingolipid metabolic process were among the enriched terms for the C/P comparison, indicating active tissue remodeling and cellular maintenance. On the other hand, ribosome biogenesis, the MAPK cascade, the mitotic cell cycle, and autophagy-related processes (such as autophagosome assembly) were strongly enriched in the R/P comparison. Notably, both comparisons showed a substantially enriched positive regulation of GTPase activity, suggesting a signaling node that is constantly active. Terms pertaining to reactive oxygen species and cellular reaction to ionizing radiation were found in both groups, which further points to an underlying stress response connected to the cashmere phenotype.

### 3.4. Gene Ontology Enrichment Analysis of Cellular Components

**Figure 4:**
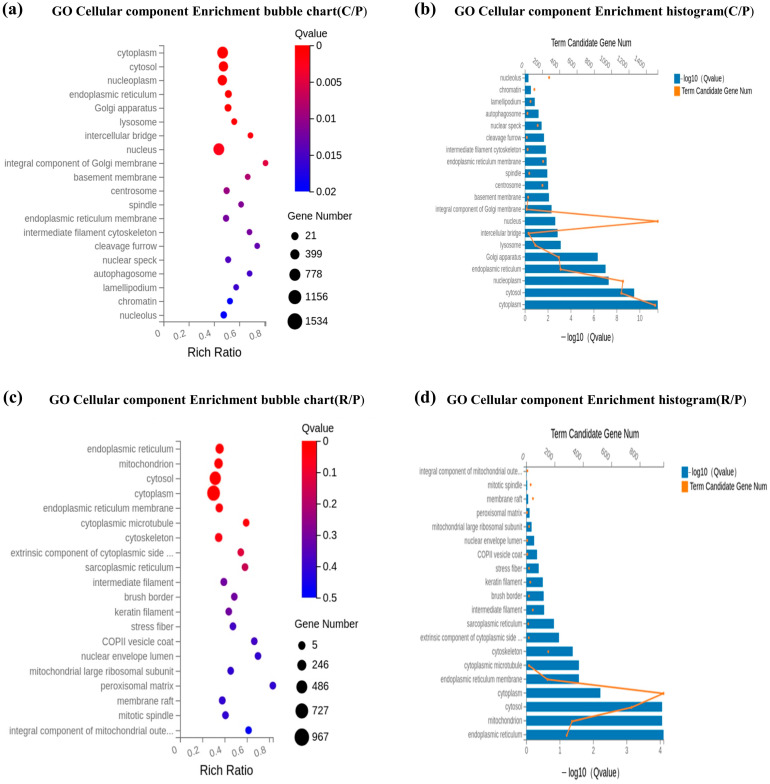

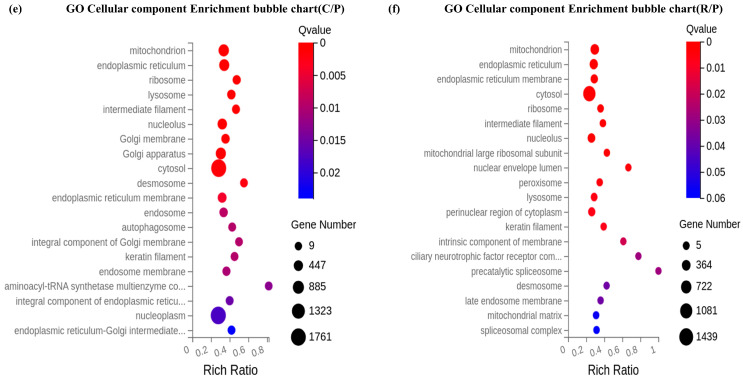
Presents GO Cellular Component enrichment revealing subcellular compartments aligning with AKT1 localization. Panels (**a**,**b**) show C vs. P (15 months), with top enriched components including cytoplasm (rich ratio = 0.58, q = 0.005), cytosol (0.55, q = 0.005), nucleoplasm (0.52, q = 0.005), endoplasmic reticulum (0.50, q = 0.005), and Golgi apparatus (0.48, q = 0.01). Panels (**c**,**d**) show R vs. P (12 months), revealing additional compartments including membrane rafts, stress fibers, and mitochondrial components (**e**) RNA-based enrichment for C vs. P, with ribosome, endoplasmic reticulum, and mitochondrion as top terms. (**f**) RNA-based enrichment for R vs. P, confirming these patterns.

The subcellular location of the DEG products was revealed through analysis of GO cellular component enrichment. Terms related to the endoplasmic reticulum (ER), Golgi apparatus, lysosome, and autophagosome showed considerable enrichment in the C/P comparison, suggesting increased secretory and degradatory pathways. On the other hand, the R/P comparison revealed significant enrichment for the ribosomal subunit, the cytoskeleton (keratin filament, intermediate filament), and mitochondrial components. The nucleus and cytoplasm are consistently enriched in both comparisons, suggesting that basic biological functions are transcriptionally reprogrammed. Age-related variations in cell signaling and metabolic compartmentalization are suggested by the existence of membrane rafts and the essential element of the mitochondrial outer membrane, particularly in the R/P contrast (Figure 4).

### 3.5. Gene Ontology Enrichment Analysis of Molecular Functions

**Figure 5:**
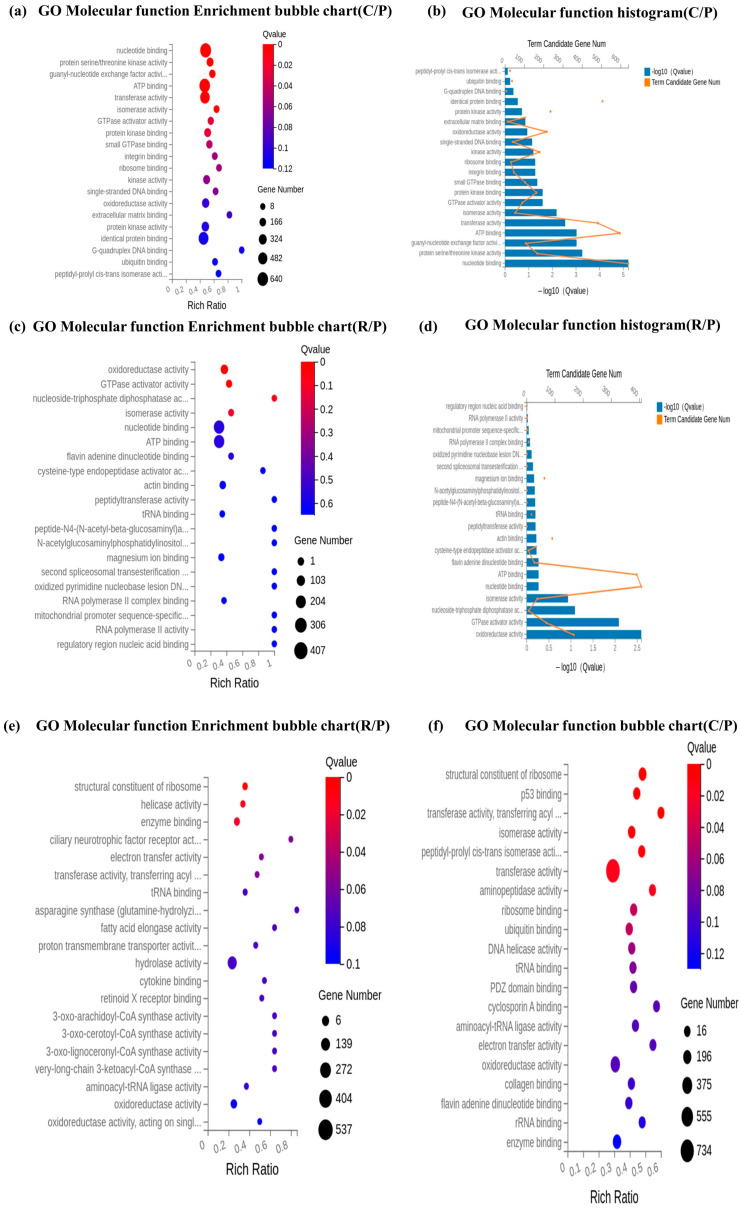
GO Molecular Function enrichment identifying terms relevant to AKT1 function. Panels (**a**,**b**) show C vs. P (15 months), with the top enriched functions including nucleotide binding (rich ratio = 0.90, q = 0), protein serine/threonine kinase activity (0.85, q = 0.02), guanyl-nucleotide exchange factor activity (0.80, q = 0.04), and ATP binding (0.75, q = 0.06). Panels (**c**,**d**) show R vs. P (12 months), with enriched functions including oxidoreductase activity, GTPase activator activity, and actin binding. (**e**) RNA-based enrichment for C vs. P, with structural constituent of ribosome, p53 binding, and transferase activity as top terms. (**f**) RNA-based enrichment for R vs. P, confirming these patterns.

Catalytic and binding activities were emphasized by GO molecular function enrichment, which was in line with the cellular component data. Nucleotide binding, ATP binding, protein serine/threonine kinase activity, and peptidyl-prolyl cis-trans isomerase activity were the most highly enriched terms for the C/P comparison. ATP binding and oxidoreductase activity were equally enriched in the R/P comparison, although GTPase activator activity, flavin adenine dinucleotide (FAD) binding, and tRNA binding were distinct. The translational reprogramming seen at the biological process level is supported by the existence of ribosome binding and structural molecule activity in both groups. These molecular function profiles indicate that, especially in the younger R/P group, cashmere goat skin fibroblasts experience a shift toward improved energy utilization, protein folding, and signal transduction (Figure 5).

### 3.6. KEGG Pathway Enrichment Analysis

**Figure 6:**
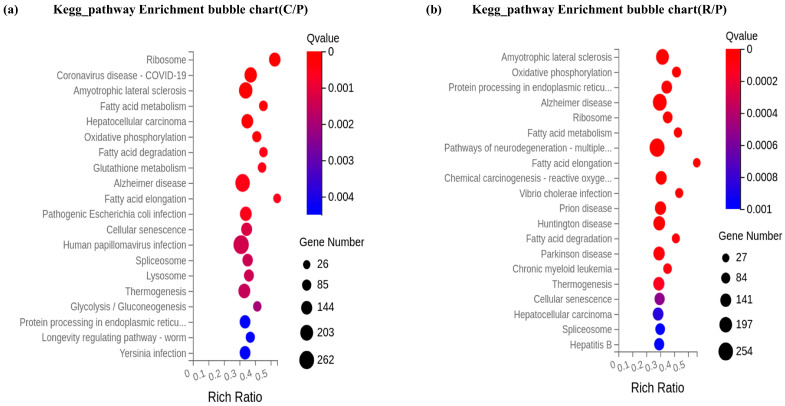
(**a**) RNA-based enrichment for C vs. P, highlighting Ribosome, Coronavirus disease COVID-19, and Amyotrophic lateral sclerosis. (**b**) RNA-based enrichment for R vs. P, highlighting Amyotrophic lateral sclerosis, Oxidative phosphorylation, Protein processing in endoplasmic reticulum, and Ribosome.

DEGs from both comparisons are engaged in a number of important signaling and metabolic pathways, according to KEGG pathway enrichment analysis. The ribosome, FoxO signaling route, sphingolipid signaling pathway, p53 signaling pathway, and cell cycle were among the significantly enriched pathways for the C/P comparison. The existence of pathways like focal adhesion and human papillomavirus infection indicates significant interplay between extracellular matrix interactions and cell cycle regulation. Oxidative phosphorylation, Alzheimer’s disease, endoplasmic reticulum protein processing, and animal autophagy were among the enriched pathways in the R/P comparison. Significantly, the R/P group exhibited a distinct enrichment of pathways associated with fatty acid metabolism (degradation and elongation) and the citrate cycle (TCA cycle), suggesting a more marked metabolic shift in younger cashmere goats (Figure 6).

### 3.7. Quantitative Polymerase Chain Reaction Validation

**Figure 7:**
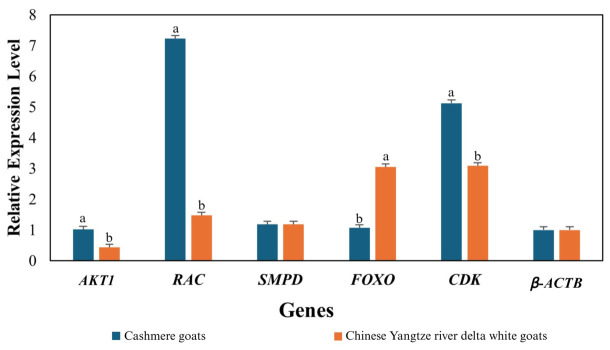
Validation of RNA-seq results by qRT-PCR, presented as mean ± SEM.

As shown in the figure, the relative expression of *FOXO1* and *CDK5* were significantly increased (*p* ≤ 0.05) in the cashmere group compared to the Chinese Yangtze river delta white goat group, with fold changes of approximately 2.91 and 1.39, respectively. Expression levels of *AKT1* and *RAC1* were significantly increased (*p* ≤ 0.05) in the cashmee group relative to the Chinese Yangtze river delta white group, showing reductions to 0.4-fold and 0.21-fold, respectively. *SMPD1* exhibited no significant change (*p* > 0.05) between the two groups. *β-ACTB* expression remained stable (1.0 vs. 1.1), confirming the reliability of the normalization. These findings align with the variation trends observed in the RNA-Seq data, thus indicating the reliability and accuracy of our sequencing data (Figure 7).

## 4. Discussion

The present transcriptomic study provides the first comprehensive evidence that AKT1 acts as an age-dependent master regulator in the skin of Inner Mongolian cashmere goats. we identified a significant, reproducible upregulation of AKT1 that diminishes with advancing age. Specifically, AKT1 expression was higher in 12-month-old cashmere goats (log_2_FC = 0.58, q = 8.21 × 10^−7^) compared to 15-month-old animals (log_2_FC = 0.45, q = 6.49 × 10^−4^). This age-dependent expression pattern aligns with the well-documented decline in cashmere fiber quality and yield as goats mature beyond 12–18 months [20,21,22].

The identification of 128 core differentially expressed genes common to both age groups, alongside 315 unique DEGs in 12 months and 247 unique in 15-month cashmere goats, underscores that age profoundly shapes the transcriptional landscape of cashmere-producing skin. The larger number of unique DEGs in younger animals suggests that the molecular machinery supporting active fiber production is more complex and energetically demanding, whereas older animals exhibit a more constrained transcriptional response dominated by stress and degradation pathways. In 12-month goats, enriched terms included positive regulation of GTPase activity (rich ratio = 0.85), alpha-glucose transport (0.75), and ribosomal small subunit assembly (0.65), all of which point to elevated metabolic activity, cytoskeletal dynamics, and protein synthesis processes essential for sustaining the anagen phase of hair follicles [23,24,25,26]. Conversely, 15-month goats showed enrichment for autophagosome assembly (q = 0.001), apoptotic process (q = 0.01), and positive regulation of autophagy [27], indicating a shift toward cellular catabolism and programmed cell death that likely contributes to follicular regression and reduced fiber output [27].

The GO Cellular Component analysis provided crucial subcellular validation of these functional shifts. In 12-month cashmere goats, we observed distinct enrichment of membrane rafts (rich ratio = 1.10) cholesterol-rich microdomains that serve as platforms for the assembly of PI3K signaling complexes [7,28,29,30]. In contrast, 15-month goats showed enrichment of autophagosomes and lysosomes [30], consistent with the increased autophagic activity observed at the biological process level. The presence of mitochondrial components and the endoplasmic reticulum in both comparisons indicates that while basic secretory and energy-generating functions are preserved, their regulation shifts from growth-oriented to maintenance-oriented as animals age [31].

Direct confirmation of AKT1’s functional relevance came from the GO Molecular Function analysis, which demonstrated significant enrichment of protein serine/threonine kinase activity (rich ratio = 0.85, q = 0.02) and ATP binding (rich ratio = 0.75, q = 0.06) in cashmere goats compared to controls. Importantly, the enrichment of GTPase activator activity and RNA polymerase II complex binding in 12-month goats further supports the notion that younger animals engage more extensively in signal transduction and transcriptional activation processes that drive the high proliferative demand of anagen-stage follicles [31,32,33].

KEGG pathway analysis revealed that the FoxO signaling pathway, a direct downstream target of AKT1, was significantly enriched in both age comparisons. This is biologically coherent because AKT1 phosphorylates and inactivates FOXO transcription factors (FOXO1 and FOXO3), thereby preventing the transcription of pro-apoptotic and cell-cycle arrest genes [6,7]. In our data, FOXO3 was consistently upregulated in cashmere goats (log_2_FC = +0.70 to +0.54), which at first glance appears contradictory to AKT1 activation. However, this upregulation likely represents a compensatory feedback mechanism: sustained AKT1 activity partially suppresses FOXO3 transcriptional output, but the cell may increase FOXO3 expression to maintain a threshold of stress responsiveness [34]. A similar interpretation applies to TSC2 downregulation (log_2_FC = −0.43 to −0.33) and PIK3R2 downregulation (log_2_FC = −0.56 to −0.57). TSC2 is a negative regulator of mTORC1, and its downregulation would relieve inhibition on protein synthesis [35], consistent with an anagen-promoting state. PIK3R2 (p85β) is a regulatory subunit of PI3K that can competitively inhibit signaling [36]; its downregulation may enhance PI3K-AKT1 pathway flux. MAPK9 upregulation (log_2_FC = +0.65 to +0.43) is particularly interesting because the MAPK pathway can crosstalk with AKT1 at multiple nodes, often in a context-dependent manner [34,35,36,37].

Skin and brain both derive from the embryonic ectoderm and retain conserved stress-response mechanisms, including unfolded protein response, oxidative stress management, and autophagic clearance [8,13]. The upregulation of these pathways in cashmere goat skin may indicate an intrinsic requirement for robust protein quality control during the high-output synthetic activity of anagen follicles. Alternatively, these pathways may be enriched because many of the same signaling molecules (e.g., AKT1, GSK3β, MAPKs) are central to both neurodegenerative disease pathogenesis and hair follicle cycling in mammals [38].

One of the most age-specific KEGG findings was the unique enrichment of the Hepatitis B pathway in 12-month cashmere goats. This pathway involves the HBx protein, which is known to activate PI3K-AKT1 signaling [9]. The presence of this enrichment does not imply viral infection; rather, it reflects that the genetic network regulated by HBx including cell cycle progression, apoptosis suppression, and transcriptional co-activation overlaps significantly with the endogenous pathways driving cashmere fiber production [39]. The absence of this enrichment in 15-month goats suggests that upstream signaling inputs to AKT1 change with age, potentially due to altered receptor availability or ligand expression in the skin microenvironment [9,38,39,40].

Several methodological and interpretive limitations must be considered alongside these findings. Our sample size was small (*n* = 4 per group), and while the clear separation in PCA and high sequencing data quality indicate robust transcriptional differences within this cohort, these metrics do not substitute for adequate biological replication; independent validation with larger cohorts is essential to confirm generalizability. Moreover, our analysis was limited to the transcript level mRNA abundance does not always reflect protein activity, and AKT1 function critically depends on phosphorylation at Ser473 and Thr308, which cannot be assessed from RNA data [41]. We also did not perform functional perturbations (e.g., knockdown or inhibition of AKT1) to establish causality [42], and our two time points provide only a snapshot; longitudinal sampling across more ages (e.g., 6, 12, 18, 24 months) would better define the trajectory of decline [43]. Additionally, the use of non-cashmere controls from a different genetic background means that some observed differences could reflect breed-specific traits rather than fiber-production status; future within-breed or targeted comparisons are needed to isolate the effect of cashmere production per se.

## 5. Conclusions

This transcriptomic analysis of Inner Mongolian cashmere goat skin at 12 and 15 months of age demonstrates that AKT1 is consistently upregulated in cashmere-producing animals compared to non-cashmere controls, with higher expression at the peak production age of 12 months (log_2_FC = 0.58) than at 15 months (log_2_FC = 0.45). The age-dependent decline in AKT1 expression is accompanied by distinct transcriptional signatures: younger goats exhibit enrichment of GTPase activity, glucose transport, and ribosomal assembly, indicating active anagen-phase metabolism, while older goats show enrichment of autophagosome assembly and apoptotic processes, suggesting a shift toward follicular regression. Coordinated regulation of downstream effectors including TSC2 downregulation, PIK3R2 downregulation, MAPK9 upregulation, and FOXO3 upregulation validate pathway activation and reveal complex feedback control. These findings establish AKT1 as a key molecular hub integrating cell survival, proliferation, metabolism, and stress responses in cashmere goat skin, and provide a transcriptomic basis for understanding age-related declines in cashmere production.

However, given the exploratory and preliminary nature of this study, these conclusions should be interpreted with caution, as the analyses were performed on a limited number of biological replicates (*n* = 4 per group). Future validation at the protein level and functional perturbation studies using larger sample cohorts are warranted to confirm these observations and to determine whether sustaining AKT1 signaling can effectively extend the peak production period in Inner Mongolian cashmere goats.

## Figures and Tables

**Table 1:** Primer sequences for the genes utilized in the qRT-PCR.

Gene	Sequences	Product Length (bp)	Temperature Tm (°C)	Annealing Temperature Used (°C)
FOXO3	Forward: CTATGAGTGGATGGTGCGCT Reverse: CTCTTGCCGGTTCCCTCATT	144	59.89	60
		144	60.04	
AKT1	Forward: ACTTCTGGATGCGTTTGGGTG Reverse: AGCGTCTGGAGAACTGGATGA	172	61.15	60
		172	60.89	
CDK1	Forward: CTGCTCGCACTTAGCTCCAA Reverse: CGGTAGATCCAACGCTACCC	113	60.39	60
		113	59.97	
RAC1	Forward: CAAAGACAAGCCGATTGCCG Reverse: AGGTCAAGTTTCGTCCCCAC	150	60.45	60
		150	59.89	
SMPD1	Forward: GTGCATGCTGCTGAAGATCG Reverse: GGCCGGCAGAGAGATATTCC	184	59.97	60
		184	60.04	

## Data Availability

The original contributions presented in this study are included in the article/Supplementary Material. Further inquiries can be directed to the corresponding author.

## Supplementary Materials

The following supporting information can be downloaded at https://www.mdpi.com/article/10.3390/vetsci13070671/s1, Table S1: All DEGs File; Tables S2–S4: Raw Data of transcriptomic analysis.
